# Dopamine Receptors and TAAR1 Functional Interaction Patterns in the Duodenum Are Impaired in Gastrointestinal Disorders

**DOI:** 10.3390/biomedicines12071590

**Published:** 2024-07-17

**Authors:** Anastasia N. Vaganova, Alisa A. Markina, Aleksandr M. Belousov, Karina V. Lenskaia, Raul R. Gainetdinov

**Affiliations:** 1 Institute of Translational Biomedicine, St. Petersburg State University, Universitetskaya nab. 7/9, 199034 St. Petersburg, Russia; a.n.vaganova@spbu.ru (A.N.V.);; 2 St. Petersburg State University Hospital, St. Petersburg State University, Universitetskaya nab. 7/9, 199034 St. Petersburg, Russia; aleksandr.belousov@spbu.ru; 3 Department of Medicine, St. Petersburg State University, Universitetskaya nab. 7/9, 199034 St. Petersburg, Russia; k.lenskaya@spbu.ru

**Keywords:** *TAAR1*, *DRD2*, *DRD4*, *DRD5*, functional dyspepsia, diabetic gastroparesis, duodenum, dopamine, trace amines

## Abstract

Currently, there is a growing amount of evidence for the involvement of dopamine receptors and the functionally related trace amine-associated receptor, TAAR1, in upper intestinal function. In the present study, we analyzed their expression in the duodenum using publicly accessible transcriptomic data. We revealed the expression of *DRD1*, *DRD2*, *DRD4*, *DRD5*, and *TAAR1* genes in different available datasets. The results of the gene ontology (GO) enrichment analysis for *DRD2* and especially *TAAR1* co-expressed genes were consistent with the previously described localization of D2 and TAAR1 in enteric neurons and secretory cells, respectively. Considering that co-expressed genes are more likely to be involved in the same biological processes, we analyzed genes that are co-expressed with *TAAR1*, *DRD2*, *DRD4*, and *DRD5* genes in healthy mucosa and duodenal samples from patients with functional dyspepsia (FD) or diabetes-associated gastrointestinal symptoms. Both pathological conditions showed a deregulation of co-expression patterns, with a high discrepancy between *DRD*s and *TAAR1* co-expressed gene sets in normal tissues and patients’ samples and a loss of these genes’ functional similarity. Meanwhile, we discovered specific changes in co-expression patterns that may suggest the involvement of TAAR1 and D5 receptors in pathologic or compensatory processes in FD or diabetes accordingly. Despite our findings suggesting the possible role of TAAR1 and dopamine receptors in functional diseases of the upper intestine, underlying mechanisms need experimental exploration and validation.

## 1. Introduction

Dopamine (DA) plays significant regulatory roles outside of the central nervous system, in several tissues, including the eyes, cardiovascular system, and endocrine pancreas [1]. The proximal gastrointestinal (GI) tract is the major source of the peripheral circulation of dopamine, especially after a meal [2]. Dopamine is released in the GI tract from the gastric parietal cells [3], enteric neurons [4], and immune cells, including macrophages, dendritic cells, and lymphocytes [5].

In adult humans, most intact fat arrives in the duodenum, where pancreatic lipase degrades it. Simultaneously, duodenal proteases act together to digest proteins. The duodenum is the site of maximal iron, zinc, and inorganic selenium absorption [6]. Gastric-derived DA protects the duodenal mucosa when gastric acid secretion increases [5,7] by stimulating bicarbonate secretion and regulating mucosal blood flow [4]. In the duodenal lumen, DA increased bicarbonate secretion via apical D2-receptor- and calcium-dependent pathways [4,7]. Also, DA enhances duodenal epithelial permeability [8] and longitudinal muscle contractions [4].

DA plays a key role in regulating GI secretory and motor activity [9], permeability [7,10], epithelium protection [4,5], and enteric neuron functioning [11]. All five dopamine receptor subtypes, i.e., D1–D5 receptors, are expressed in the GI tract [12]. Both neurons and non-neuronal cells express D1, D3, and D5 receptors, while D2 receptor expression is restricted to glial cells and neurons, where dopamine plays a role in modulating cholinergic neurotransmission [13,14]. D1 receptor activity mediates dopamine-induced relaxation, characterized by reducing the spontaneous contraction amplitude [13,14]. The D4 receptor is confined to the mucosal layer [4]. Lowered DA concentrations and DA receptor expression deregulation in the gut promote inflammation, increasing intestinal motility and heightening visceral sensitivity [10,13,15]. *DRD1* and *DRD2* double-knockout genes in mice lead to major structural and functional abnormalities in the development and function of the GI tract [16].

Trace-amine-associated receptors (TAARs) recognize both trace amines and some other amine compounds [17]. These receptors, especially TAAR1, which takes part in dopamine signaling regulation [18], are considered prospective targets for the new generation of antipsychotic drugs [19,20,21]. Currently, several TAAR1 ligands have been identified, including endogenous 3-iodothyronamine [22], β-phenylethylamine [23], tryptamine, and octopamine [17]. In the GI tract, TAAR1 is an integrator of metabolic control, which acts on gastrointestinal and pancreatic islet hormone secretion [24]. This receptor was identified in the duodenal mucosa enteroendocrine cells containing chromogranin A, GLP-1, or peptide YY and suggested regulating the secretion hormones and, consequently, a reduction in food consumption and delayed gastric emptying [24,25,26].

The effects of TAAR1 ligands on GI function and metabolism depend on their local effects in the gut and the activation of homeostatic and hedonic feeding centers [25]. Sub-chronic administration of the TAAR1 agonist Ulotaront in rats reduces body weight, food intake, and liver triglycerides compared to vehicle controls [25]. TAAR1 binding with its natural ligands like octopamine delayed the gastrointestinal transit by the relaxing effects inhibiting cholinergic stimulation [27]. Instead, *Ruminococcus gnavus*, a producer of phenethylamine and tryptamine, directly stimulates serotonin biosynthesis in intestinal enterochromaffin cells via the TAAR1-involving mechanism and exhibits pro-inflammatory properties by producing inflammatory polysaccharides [28].

In this regard, dopamine and trace amine signaling in the GI tract seems to be complex and multidirectional. The drugs used for symptom relief in dyspeptic diseases like diabetic gastroparesis or functional dyspepsia (FD) usually include prokinetics and antiemetics [27,29], including antiemetics that demonstrate an antagonistic effect on D2 receptors such as metoclopramide or domperidone [29,30,31]. However, domperidone was not approved by the FDA and has only limited availability in Europe, and only short-term use is recommended because of the risks associated with administration [32,33]. Levosulpiride is an atypical dopamine antagonist anti-psychotic drug that may be effective in FD [33], especially taking into account that the development of FD was nearly 8-fold higher in those with baseline anxiety [34].

Hence, the aim of the present study is the evaluation of the expression and functional significance of trace amine-associated receptors, including the prospective drug target for neuropsychiatric diseases, TAAR1 [35], and dopamine receptor expression in the normal duodenum and in diseases that involve the duodenal part of the intestine.

## 2. Materials and Methods

### 2.1. Public Resources and Databases

The expression data were obtained from the public database of GEO [36] from NCBI and Expression Atlas from EBI [37]. We used the terms “duodenum” and “duodenal”. The inclusion criteria were: (1) *TAAR* and *DRD* expression values are available; (2) expression data are available in raw counts; (3) at least 5 duodenal samples from each study group are available; and (4) the datasets represent the expression profiles of native human samples; data for organoids were excluded.

Two independent reviewers (A.N.V. and A.A.M.) extracted data from the included datasets and performed the statistical analysis. The results were cross-checked by A.N.V. and A.A.M., and disagreements were resolved by reinvestigation.

### 2.2. Data Normalization and Statistical Analysis

Raw counts for datasets were downloaded from the GEO NCBI repository. Data were in count per million (CPM) normalized by the edgeR package (version 4.0.16) [38]. CPM values above the threshold level of 0.1 were considered positive. Expression data were visualized by the ggplot2 package (version 3.5.1) [39].

Differentially expressed genes were identified by the likelihood ratio test using the edgeR package [38]. The *p*-values were adjusted for multiple testing corrections by the Benjamini–Hochberg method. If the adjusted *p*-values (Padj) were less than 0.05, we considered the genes to be differentially expressed.

### 2.3. Measurement of Co-Expression and GO Enrichment Analysis

*TAAR1, DRD2, DRD4*, and *DRD5* co-expressed genes were selected by Spearman’s correlation coefficient (ρ > 0, *p* < 0.05). The GO enrichment analysis (identification of GO terms that are significantly enriched by the genes of the selected set) was performed in the identified co-expressed gene clusters, and visualization of the results was performed by the clusterProfiler Bioconductor package (version 4.10.1) [40]. We considered significant enrichment results only for GO biological process terms with a false discovery rate value of <0.05. 

### 2.4. Analysis of Functional Semantic Similarity between Genes

Gene ontology (GO) semantic similarity was calculated by Wang’s method [41] in the GOSemSim (version 2.28.1) [42] package employing the “Biological process” GO terms [43,44]. To compare semantic similarity scores in different gene clusters, we used the Wilcoxon test and compared them with the results from the same analysis on a random gene set.

## 3. Results

### 3.1. Data Search, Selection, and Inclusion

**Figure 1:**
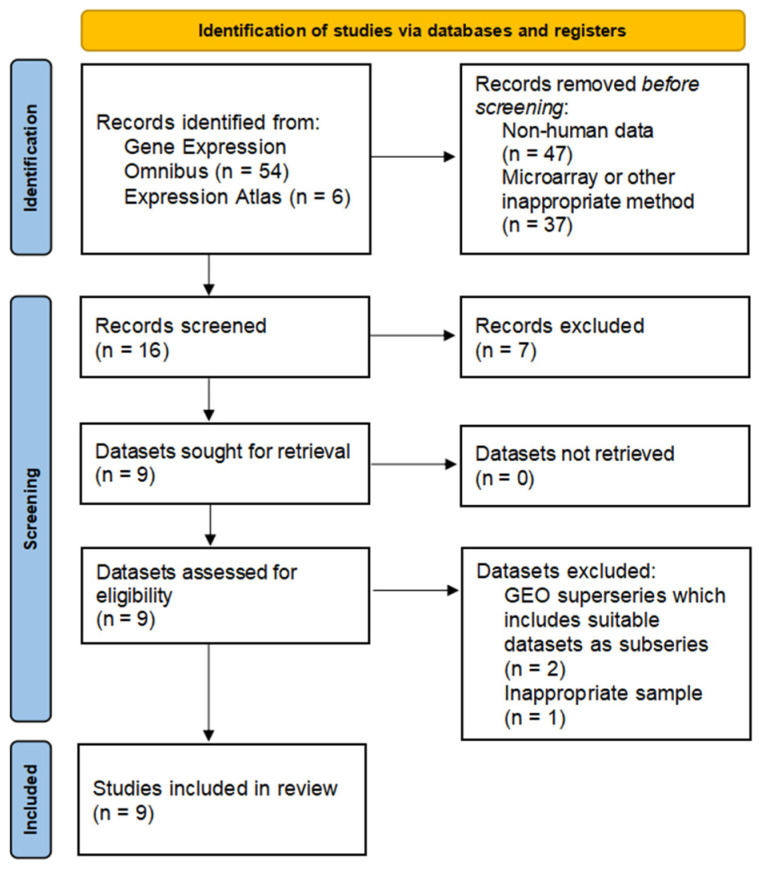
Flowchart of the data search.

We reviewed all datasets, including data for gene expression in the duodenum. An initial search identified 60 records. The applied selection criteria reduced the number of datasets to six datasets from the GEO repository (Figure 1).

The key characteristics of the studies are described in Table 1.

### 3.2. Dopamine Receptor Genes and TAAR mRNA Expression in the Healthy Duodenum

**Figure 2:**
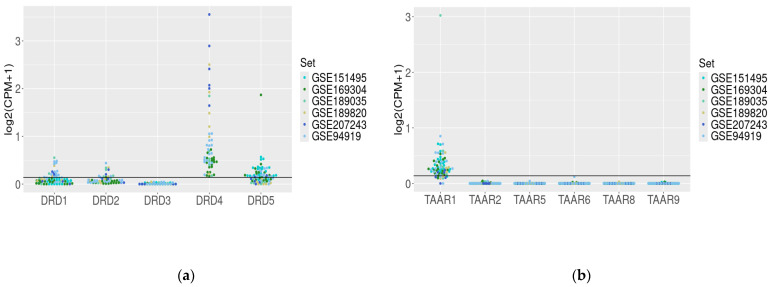
*DRD* (**a**) and *TAAR* (**b**) gene mRNA expression in the duodenum biopsy samples from healthy subjects (GSE151495, GSE169034, GSE189820, and GSE207243) and non-affected duodenal samples from patients with familial adenomatous polyposis (GSE94919, GSE189035).

To estimate the dopamine and trace-amine-associated receptor expression in the healthy duodenal wall, we analyzed six transcriptome RNA-generated datasets. The extracted data demonstrated congruent *DRD* (except *DRD3* mRNA) genes and *TAAR1* gene expression in different datasets, representing the expression patterns in duodenal samples from healthy subjects (GSE151495, GSE169034, GSE189820, and GSE207243) and non-affected duodenal samples from patients with familial adenomatous polyposis (GSE94919, GSE189035, Figure 2a,b).

For further analysis, we chose the most expressed genes, which included *DRD4* (100% of healthy duodenal mucosa samples), *DRD5* (58% of samples), and *TAAR1* (85% of samples). Additionally, regarding the literature data that describe D2 receptor expression in the duodenum [4,8,30] and its interaction with TAAR1 [18], we also included its gene, *DRD2*, in the analysis despite the low frequency of identifiable expression (25%). *DRD1* gene mRNA was identified in 26% of samples. The *DRD1* gene was identified in 26% of samples. There were no positive samples for *DRD3* or *TAAR2*–*TAAR9* expression found in the healthy duodenal mucosa biopsies.

### 3.3. Dopamine Receptor Genes and TAAR1 mRNA Co-Expressed Clusters in Healthy Duodenum Demonstrate High Heterogeneity

**Figure 3:**
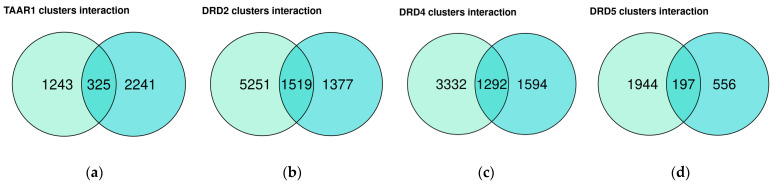

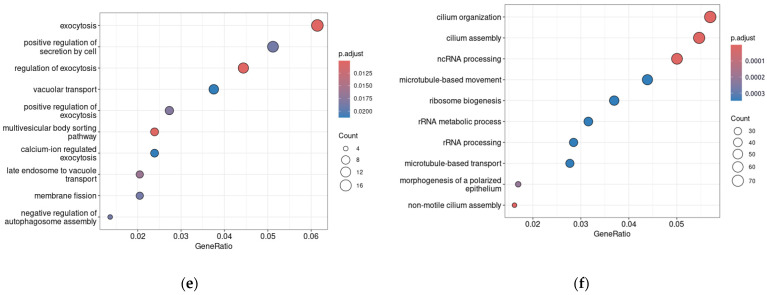
Functional analysis of TAAR1 and DRD co-expressed genes in the healthy duodenal mucosa. Venn diagrams illustrate TAAR1 (**a**), DRD2 (**b**), DRD4 (**c**), and DRD4 (**d**) overlay in GSE151495 (marked by aquamarine green) and GSE169304 (marked by cyan blue). Gene ontology (GO) biological process (BP) enrichment analysis of genes co-expressed with TAAR1 (**e**) and DRD2 (**f**) in both datasets. GO biological process terms showed no appropriate enrichment in the DRD4 and DRD5 co-expressed gene cluster.

Two datasets included in the review, i.e., GSE151495 and GSE169304, comprise groups of duodenal samples from healthy subjects, which consist of more than 10 samples. For these datasets, we identified genes that are co-expressed with *TAAR1*, *DRD2*, *DRD4*, or *DRD5* genes (ρ > 0, *p* < 0.05). The lengths and overlapping of the identified clusters are depicted in Figure 3a–d.

We performed a GO BP term enrichment analysis in the gene subsets, which includes genes co-expressed with *TAAR1*, *DRD2*, *DRD4*, or *DRD5* in both GSE151495 and GSE169304 datasets, to explain their biological function. No significant GO term enrichment results were revealed in the genes co-expressed with *DRD4* or *DRD5*. In contrast, the *TAAR1* gene is co-expressed with genes associated predominantly with exocytosis and secretion (Figure 3e). Despite the previously described functional interaction between TAAR1 and D2R [18], *DRD2* is co-expressed with genes involved in microtube organization and RNA processing (Figure 3f).

### 3.4. Dopamine and Trace Amine Co-Expression Patterns in the Duodenum Are Disrupted in Patients with Functional Dyspepsia

The GSE169304 dataset includes gene expression data for duodenal samples of healthy donors (*n* = 18) and patients with FD (*n* = 37). Age-matched FD and control groups were included in the study according to the design description in the paper, which describes the dataset. The mean age was 42 years for FD and 40 years for healthy subjects. Groups also were matched for body mass index (BMI); the mean values were 26.7 kg/m^2^ and 26.1 kg/m^2^ in the FD and control groups, respectively, and included 78% and 58% female subjects for the FD and control groups accordingly [45]. The expression of *DRD2*, *DRD4*, *DRD5*, and *TAAR1* genes was detected in both study groups. No significant differences were identified in selected gene expression levels in healthy subjects’ mucosa and samples from FD patients.

**Figure 4:**
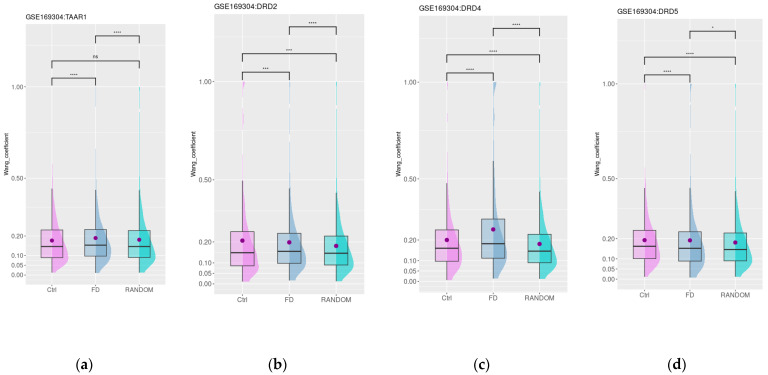
Functional similarity of *TAAR1* (**a**) and *DRDs* (**b**–**d**) co-expressed genes in duodenal mucosa from healthy donors and patients with functional dyspeptic disorders. Ctrl—healthy donors, FD—functional dyspepsia, RANDOM—random gene set, *—*p* < 0.05, ***—*p* < 0.001, ****—*p* < 0.0001, n.s.— non-significant.

Then, we estimated the functional similarity of genes that are the most (i.e., top 250) co-expressed with *TAAR1* and *DRD*s in healthy controls or in patients with FD. The functional similarity of genes co-expressed with *DRD*s in healthy controls and patients with FD was higher than in a random 250-gene set (*n* = 250, Figure 4b–d). The number of common genes highly co-expressed with *DRD*s both in healthy subjects and FD patients was low (eight genes for *DRD2*, nine genes for *DRD4*, and three genes for *DRD5*). In contrast, only for FD patients’ samples, *TAAR1* co-expressed genes’ semantic similarity was higher than in the random genes set (Figure 4a) and the number of common genes highly co-expressed with *TAAR1* both in healthy subjects and FD patients stood at 28.

For *DRD2* and *DRD5* co-expressed genes, mean semantic similarity values were significantly lower in the FD samples compared to healthy subjects’ mucosa (Figure 4b,d) and for *TAAR1* and *DRD4* co-expressed genes, the mean semantic similarity became higher in the FD patients’ samples. The growth of semantic similarity between *TAAR1* co-expressed genes in FD-patient-derived samples may mirror the identified switch from the co-expression of *TAAR1* with genes involved in the response to glucose in healthy subjects to genes attributed to more consolidated GO terms characterizing γ-aminobutyric acid (GABA) signaling in FD patients (Supplementary Figure S1). *DRD4* co-expressed genes did not show any significant GO enrichment results in both healthy mucosa and FD samples.

### 3.5. Dopamine and Trace Amine Co-Expression Patterns in the Duodenum Are Impaired in Patients with Gastrointestinal Disorders Associated with DM

**Figure 5:**
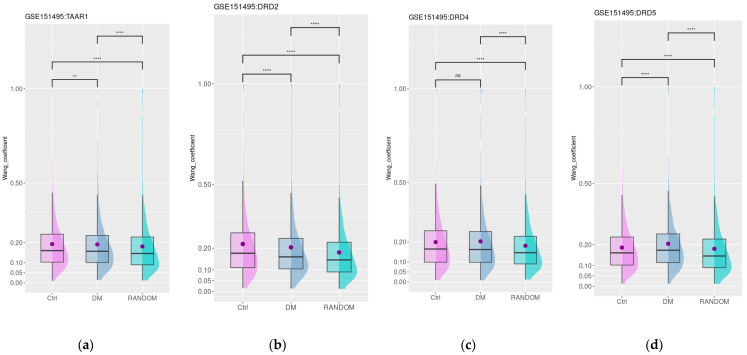
Functional similarity of *TRAA1* (**a**) and *DRD* (**b**–**d**) co-expressed genes in duodenal mucosa from healthy donors and patients with diabetes mellitus. Ctrl—healthy donors, DM—diabetes mellitus, RANDOM—random gene set, **—*p* < 0.01, ****—*p* < 0.0001, n.s.— non-significant.

The impact of diabetic gastrointestinal complications on the duodenal mucosa was estimated in the GSE151495 dataset, which includes gene expression data for duodenal samples of healthy donors (*n* = 21) and patients with DM and associated gastrointestinal symptoms (DM, *n* = 39). The age, sex distribution, and BMI were not significantly different between groups, as specified by the dataset authors in the corresponding article. The mean age was 45 and 40 years for DM and healthy subjects, the mean BMI was 26 and 28 kg/m^2^, and 58% and 78% of subjects were female in the specified groups, respectively [46]. The expression of *DRD2*, *DRD4*, *DRD5*, and *TAAR1* was not found to be significantly different between the mucosa of healthy individuals and samples from individuals with diabetes mellitus. Then, we estimated the functional similarity of genes most co-expressed (i.e., top 250) with *TAAR1* and *DRDs* in healthy controls and patients with DM. Only a low number of common genes were identified in these groups (8 genes for *TAAR1*, 5 genes for *DRD2*, 48 genes for *DRD4*, and 15 genes for *DRD5*). For all four groups of co-expressed genes, the mean semantic similarity Wang values were higher than in the random gene set (*n* = 250, Figure 5a–d). Additionally, for *DRD2* functionally connected with *TAAR1*, the mean semantic similarity values were slightly but significantly lower in the diabetes mellitus samples compared to healthy subjects’ mucosa. This loss of functional links may be related to the disruption of D2R- and TAAR1-mediated biologic processes in diabetic-associated islet damage (Figure 5a,b).

In contrast, for *DRD4*, the co-expression landscape remains relatively stable (Figure 3c), and functional relations between genes highly co-expressed with *DRD5* in DM patients seem to be more strengthened than in healthy controls (Figure 3d). The GO enrichment analysis demonstrated the switch of the functional role of *DRD5* co-expressed genes from the participation of different secretion-related biological processes in healthy subjects to the single GO term “response to hydrogen peroxide” in DM patients (Supplementary Figure S2).

## 4. Discussion

From the perspective of the emerging data suggesting the role of dopamine signaling in normal duodenal functioning and the development of pathological conditions- [4,5,7,9,10,14,47], we analyzed dopamine receptors’ gene expression and their association with biological process activity in the duodenal mucosa. Additionally, we evaluated the expression of the *TAAR1* gene coding the modulator of dopamine transmission and the prospective drug target in the duodenal samples. Further, we estimated the stability of expression and functional associations of these genes in the duodenal mucosa from subjects suffering from functional gastrointestinal disorders or FAP. First, we identified dopamine receptor gene (excluding *DRD3*) expression in the normal duodenal samples, consistent with the current literature data [7,24,26,30].

To evaluate the functional significance of TAAR1 and dopamine receptors in the duodenal mucosa, the genes co-expressed with *TAAR1* and *DRD* genes were selected and analyzed by the GO terms enrichment method. The *DRD2* gene is co-expressed with two major functional groups. The first of these groups is related to cilia organization and the second is associated with synthetic and regulatory activity, i.e., rRNA or ncRNA processing, and ribosome biogenesis. Considering previously revealed *DRD2* expression in the enteric neurons [4,11,13], the defined associations may be interpreted in the context of D2R involvement in the activity of these cells. Cilia were identified in differentiated neurons of the enteric nervous system [48] and the damage of enteric neuron ciliation may be associated with neurodegenerative diseases [49]. Meanwhile, the co-expression of *TAAR1* with genes involved in the secretory processes is in agreement with the previously described TAAR1 expression in the secretory cells of the GI tract [24,50]. At the same time, the estimation of the functional significance of genes co-expressed with *DRD4* and *DRD5* genes did not demonstrate any specific enrichment, possibly due to the complex expression patterns of these genes. At least, it is known that the D5R receptor presents both in neuronal and non-neuronal cells in the intestinal wall [4].

To identify the stability of this molecular milieu in damaged duodenal tissues, we included four available datasets, which represent the transcriptomic data for duodenal samples both for healthy subjects and patients with dyspeptic disorders related to DM or other unidentified reasons (i.e., FD subjects).

FD is a clinical syndrome with postprandial satiety, upper abdominal pain, or burning sensations that cannot be explained by other pathologically based disorders [51,52]. *DRD2* TaqI polymorphism is associated with stress exposure, and may also be involved in the development of FD. Furthermore, the D2 receptor antagonists like domperidone [51], itopride, and sulpiride alleviate dyspeptic symptoms in FD patients [9]. We analyzed the FD impact on TAAR1 and dopamine receptors’ functional interactions in the dataset GSE169304, in which FD was associated with modest downregulation of the expression of several barrier proteins, including tight junction proteins, adherens junction proteins, and desmosomal proteins [51]. We identified the association of *TAAR1* expression with several GABA receptors in this pathologic condition. The primary function of GABA receptors, at least GABA_A_ and GABA_C_, is the regulation of duodenal motility [52]. The abnormal duodenojejunal motility is one of the mechanisms in complex FD pathophysiology [53]. In addition, γ-aminobutyric acid drugs may significantly improve the quality of life of FD patients [54]. On this background, it may be speculated that TAAR1 ligands may demonstrate an ameliorative effect on FD like anxiolytics, as the anxiolytic compounds may be effective in the treatment of FD [55], and modulators of D2R dopamine receptor activity.

Up to 50% of patients with DM have delayed gastric emptying, which is associated with hyperglycemia, enteric neuromuscular inflammation [44,55], loss of Cajal’s interstitial cells and enteric glial cells, and dysfunction in the sympathetic or parasympathetic nervous systems [27,44,55]. These symptoms may be ameliorated with antiemetics, including dopamine receptor antagonists like metoclopramide or domperidone [29].

Previously, the analysis of the GSE151495 dataset identified pronounced differences in the duodenal transcriptomes between DM patients and healthy subjects, characterized by a loss of mitochondrial DNA-coded gene expression accompanied by up-regulated nuclear DNA-coded mitochondrial gene expression. These features correlated with neuropathy and more prolonged gastric emptying [45]. The identified association of *DRD5* expression and genes involved in the response to hydrogen peroxide may mirror the participation of the D5Rreceptor in some pathogenesis-associated or compensatory processes. Furthermore, as was identified previously, the D5R receptor mediates a decrease in mitochondrial reactive oxygen species production [56] and oxidative stress [57] in the kidney. The GSE151495 dataset includes samples from both patients with type 1 diabetes and patients with type 2 diabetes [46], so the identified functional associations in patient samples are associated with DM-related hyperglycemia rather than with pathologic changes specific for specific DM types.

The co-expression cluster analysis demonstrates the pronounced changes in the functional relationship of *DRDs* and TAAR1s with other genes, mostly manifested in the restructuring of co-expression landscapes and weakening of the functional relationships between co-expressed genes. Meanwhile, we identified some associations that may mirror the association of DA-mediated signaling with some compensatory processes both in FD and DM-associated gastrointestinal symptoms. Hopefully, further research will clarify whether these intuitions are correct.

The represented results must be seen with some limitations. (i) Only a few datasets with the limited study groups in the GEO [36] repositories were relevant to the study. To overcome this limitation, we try to demonstrate the reproducibility of results of the different datasets, which were obtained independently. (ii) The mRNA abundance does not fully reflect receptor expression and activity, which also depends on post-transcriptional processes including RNA stability, modifications, translation rate and protein turnover, and molecular context. (iii) The analyzed data were received by the RNA sequencing in complex tissue samples, in which the studied genes are non-homogeneously expressed in specific cell populations, which may mask some expression differences and relationships. (vi) The comparative study of GO semantic similarity levels in selected gene sets (including gene clusters identified by correlation analysis) was described previously [58,59,60]. It provides information on the functional relevance of identified associations. On the other hand, the opportunities for more detailed analysis of common gene functions were limited in this study, possibly because of samples’ complex structures and population heterogeneity. (v) Finally, our study lacks experimental validation. However, *DRDs* and *TAAR1* expressions were previously described by diverse approaches and published by different research groups [4,7,8,26], so the present study concentrates on the description of its functional associations and their stability. Further studies using high-throughput methods and larger groups are now needed for the elucidation of the underlying mechanisms.

## 5. Conclusions

Six independent public RNAseq datasets demonstrate that healthy duodenal samples exhibit pronounced gene expression of *TAAR1*, *DRD4*, and *DRD5*. Moreover, we identified the gene expression of *DRD1* and *DRD2* in healthy duodenal mucosa, albeit at lower levels. GO enrichment analysis, following current data for the expression of *TAAR1* in GI secretory cells, demonstrates TAAR1′s association with secretory processes. Also, for *DRD2* expression, which has previously been identified in enthetic neural systems, GO enrichment analysis demonstrated the association with biological processes that may be interpreted in the context of neuron functioning. We did not receive any GO enrichment results for *DRD4* and *DRD5* genes, possibly due to their complex expression patterns. We did not identify differences in the expression levels of dopamine receptors or TAAR1 in healthy subjects and patients with FD or DM. Meanwhile, the functional associations of dopamine receptors and TAAR1 seem to be disrupted. First, the majority of genes were pronouncedly co-expressed with *TAAR1* and *DRD2*, *DRD4*, or *DRD5* in normal tissues, lose these associations in FD or DM patients. The functional relationships between genes become co-expressed with *TAAR1* and *DRD2, DRD4* or *DRD5* in DM and FD patients’ mucosa is weaker than in healthy subjects. On the other hand, we identified the association between GABA receptors and TAAR1 in the duodenal mucosa from FD patients, which is not typical for tissues from healthy subjects. Also, the association of *DRD5* and genes involved in the response to hydrogen peroxide was identified in DM patients’ duodenal samples. Since DM duodenal tissues experience oxidative stress and the D5R receptor has previously been identified as the regulator of the oxidative stress response in kidney tissue, this association may reflect the contribution of D5R to compensating for DM-related duodenal impairment. 

## Figures and Tables

**Table 1:** RNAseq datasets included in the analysis.

Dataset ID	Title	*n*	Samples Characteristics
GSE94919	Chemoprevention with COX2 and EGFR inhibition in FAP patients: mRNA signatures of duodenal neoplasia	17 ^1^	Uninvolved duodenal tissue from familial adenomatous polyposis patients
GSE151495	Altered duodenal mucosal mitochondrial gene expression is associated with delayed gastric emptying in diabetic gastroenteropathy [mRNA-Seq]	21	Duodenal mucosa from healthy subjects
		39	Duodenal mucosa from diabetes mellitus patients
GSE169304	Duodenal mucosal barrier in functional dyspepsia [mRNA]	18	Duodenal mucosa from healthy subjects
		37	Duodenal mucosa from patients with functional dyspepsia
GSE189035	PIGA mutation and glycosylphosphatidylinositol anchor dysregulation in polyposis-associated duodenal tumorigenesis	10 ^1^	Uninvolved duodenal tissue from familial adenomatous polyposis patients
GSE189820	Gene expression in duodenal biopsy samples from CVID enteropathy patients and healthy controls	6 ^1^	Duodenal mucosa from healthy subjects
GSE207243	Duodenal inflammation in common variable immunodeficiency has altered transcriptional response to viruses	5 ^1^	Duodenal mucosa from healthy subjects

^1^ The number of samples included in the present study.

## Data Availability

All datasets are available in the GEO database (https://www.ncbi.nlm.nih.gov/geo/, accessed on 14 April 2024).

## Supplementary Materials

The following supporting information can be downloaded at: https://www.mdpi.com/article/10.3390/biomedicines12071590/s1, Figure S1. Analysis of gene ontology (GO) enrichment of DRD5 co-expressed gene clusters in duodenal mucosa from healthy subjects and patients with diabetes mellitus. Figure S2. Analysis of gene ontology (GO) enrichment of TAAR1 co-expressed gene clusters in duodenal mucosa from healthy subjects and patients with functional dyspepsia.
