# Supplementary material for: Dopamine Receptors and TAAR1 Functional Interaction Patterns in the Duodenum Are Impaired in Gastrointestinal Disorders
Source: Biomedicines. 2024 Jul 17;12(7):1590. doi: 10.3390/biomedicines12071590 (PMC11274761; doi:10.3390/biomedicines12071590)
Supplement: Supplementary file 1 [file biomedicines-12-01590-s001.zip › biomedicines-3049930-supplementary.pdf]

SUPPLEMENTARY

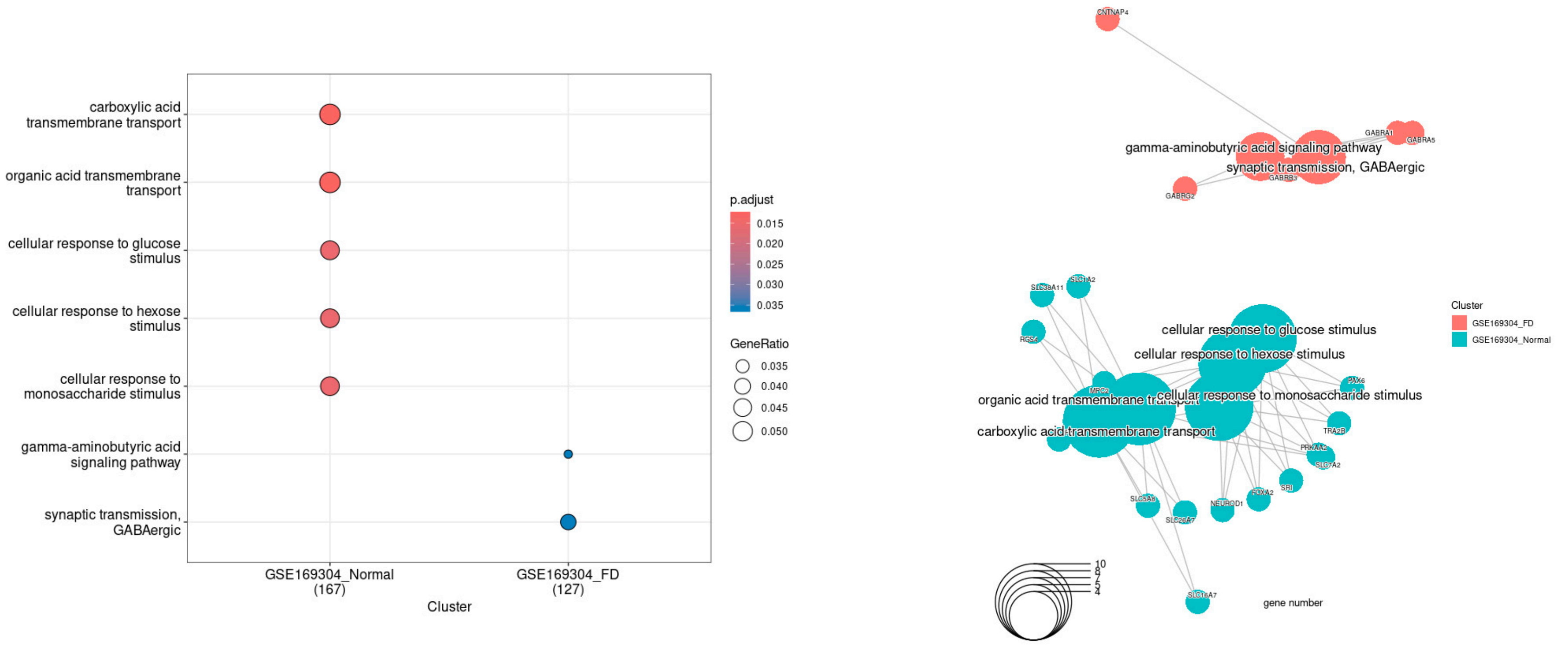

(a) **(b)**

Figure S1. Analysis of Gene Ontology (GO) enrichment of *TAAR1* co-expressed gene clusters in duodenal mucosa from healthy subjects (Normal) and patients with functional dyspepsia (FD) represented by dotplot **(a)** and cnetplot **(b)**.

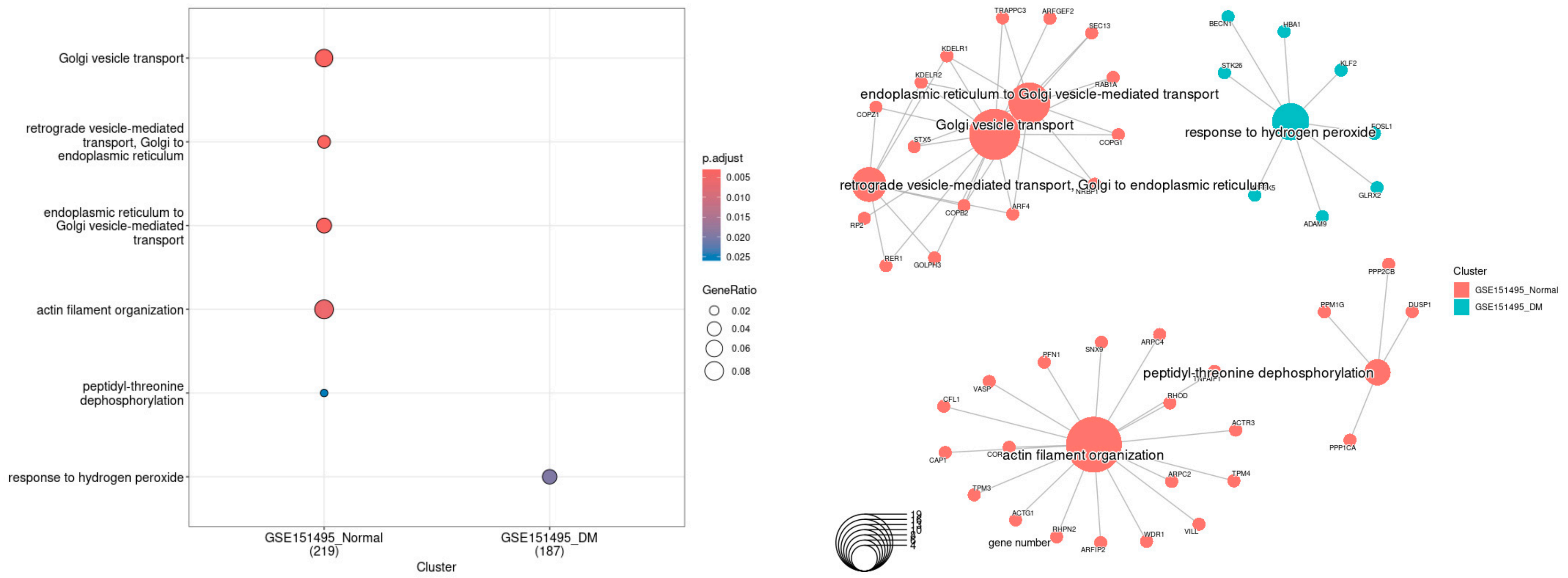

(a) **(b)**

Figure S2. Analysis of Gene Ontology (GO) enrichment of *DRD5* co-expressed gene clusters in duodenal mucosa from healthy subjects(Ctrl) and patients with diabetes mellitus (DM) represented by dotplot **(a)** and cnetplot **(b)**.
